# Practical Advice for Emergency IUD Contraception in Young Women

**DOI:** 10.1155/2015/986439

**Published:** 2015-07-29

**Authors:** Norman D. Goldstuck, Dirk Wildemeersch

**Affiliations:** ^1^Department of Obstetrics and Gynaecology, Faculty of Medicine and Health Sciences, Stellenbosch University and Tygerberg Hospital, Western Cape, South Africa; ^2^Gynecological Outpatient Clinic and IUD Training Center, Rooseveltlaan 43/44, 9000 Ghent, Belgium

## Abstract

Too few women are aware of the very high efficacy of intrauterine copper devices (IUDs) to prevent pregnancy after unprotected intercourse. Women who frequently engage in unprotected intercourse or seek emergency contraception (EC) are at high risk of unplanned pregnancy and possible abortion. It is therefore important that these women receive precise and accurate information about intrauterine devices as they may benefit from using an IUD for EC as continuing contraception. Copper IUDs should be used as first choice options given their rapid onset of action and their long-term contraceptive action which require minimal thought or intervention on the part of the user. In the United States, there is only one copper IUD presently available which limits treatment options. There are numerous copper IUDs available for use in EC, however, their designs and size are not always optimal for use in nulliparous women or women with smaller or narrower uteruses. Utilization of frameless IUDs which do not require a larger transverse arm for uterine retention may have distinct advantages, particularly in young women, as they will be suitable for use in all women irrespective of uterine size. This paper provides practical information on EC use with emphasis on the use of the frameless IUD.

## 1. Emergency Contraceptive Methods

Most women are aware of the existence of emergency contraceptive pills; however, many do not know their mechanism of action, when or how to take them, and their overall effectiveness and may inadvertently rely on them as their principal means of contraception. Given the lack of information available on both the use of copper IUDs as EC and its long-term contraceptive benefits, it is safe to assume that the knowledge of both patients and physicians on the benefits of copper releasing IUDs is even lower.

## 2. Oral Emergency Contraceptives

There are two available oral methods for EC, 1.5 mg levonorgestrel (LNG) (e.g., Plan B, Norlevo and Levonelle) and 30 mg ulipristal acetate (UPA) (e.g., Ella and EllaOne). They work through delaying ovulation and are effective up to 5 days after unprotected intercourse although the efficacy of LNG decreases close to the time of ovulation. Once ovulation has occurred, EC pills are likely to be ineffective to prevent pregnancy. The overall efficacy for women taking oral EC during their fertile window (from 5 days before ovulation to 1 day after ovulation) was 60–68% in two studies evaluating these methods [1, 2]. Taking oral EC after unprotected intercourse prior to the fertile period appears to be optimal. The pregnancy rates were 4 times higher in women taking oral LNG or UPA EC who had unprotected intercourse the day prior to ovulation compared to those who had sex outside the fertile period [3].

Oral EC has the advantage of being easily obtainable although the cost may be expensive. There are also disadvantageous which should be realized as the oral EC methods have a higher pregnancy rate in women who have unprotected sex in the fertile window. EC pills also appear to be less effective in overweight women, especially LNG, with a pregnancy risk in the higher weight categories (women weighing between 70 and 80 kg or more and body mass index (BMI) over 35) similar to expected rates in the absence of contraception [4]. Ulipristal acetate may be considered in these women as the impact of BMI appears less pronounced than with LNG [2]. Repeated acts of unprotected intercourse also appear to be a risk factor [2]. Furthermore, some drugs (e.g., anticonvulsants, antituberculosis drugs) may reduce the concentration of levonorgestrel and ulipristal. Ulipristal should not be used in women taking drugs that can reduce its absorption (e.g., antacids, H2 receptor antagonists, and proton pump inhibitors) or reduce its systemic concentration by inducing liver enzymes [5].

## 3. Intrauterine Devices

### 3.1. Effectiveness of Copper IUDs for Emergency Contraception

Oral EC methods are useful to prevent pregnancy after unprotected intercourse but they do not contribute to lowering the number of future unintended pregnancies and induced abortions [7]. Copper-releasing IUDs, on the other hand, are not only more effective than oral EC methods, but they also contribute significantly to reducing future unintended pregnancies and abortions. Copper ions are toxic to sperm and to the ovum. They alter motility and function of sperm and ova and cause alterations in the uterus and oviducts. As such, they prevent fertilization. When inserted after ovulation, they usually prevent implantation [8].

Copper IUDs have three main advantages over oral EC. (1) The efficacy of copper IUDs has been clearly established. A recent systematic review of 42 studies reported a pregnancy rate of 0.09% which is 10 times better than oral ECs [9]. (2) Currently, it is recommended that a copper IUD can be inserted up to 5–7 days after unprotected intercourse or up to 5 days after the earliest estimated day of ovulation. In this situation, the copper IUD may act by preventing implantation; when used in the usual manner, it usually prevents fertilization [10]. For the sake of clarity, in the event when sex occurred more than 5 days prior to the subject presenting, but the expected ovulation date was 5 days or less than 5 days ago, a copper IUD can still be inserted because of its preimplantation effect as implantation may occur only 6 days after ovulation [11]. In line with this, official guidelines by WHO and other organizations (American College of Obstetricians and Gynecologists (ACOG) and the Royal College of Obstetricians and Gynaecologists of the United Kingdom) advise that copper IUDs be used within 5 days of unprotected intercourse in order to avoid IUD insertion after implantation which is viewed as the start of pregnancy. However, this viewpoint has no scientific basis. It is based on the assumption that insertion of an IUD at the time or a day or more after possible implantation is forbidden. There are two reasons for this. The first is that disrupting a newly implanted conceptus is viewed as not being morally correct. However, this is a philosophical problem not a scientific one [12]. The second is that, in the same way as an IUD may predispose to infection during an established intrauterine pregnancy where it remains* in situ*, the same may analogously be true for a pregnancy of only a few cells in size and which is only a few days old. There is no evidence at all for this prejudicial approach. In fact, there is evidence that IUDs as EC are effective and safe after this time [13] and that they may be inserted at any time of the cycle if a high sensitivity pregnancy test is negative [14]. This approach is evidence based and reduces possible errors in that the subject does not have to accurately remember the day of last menstrual period or the day(s) of unprotected coitus and makes the decision to insert an emergency IUD more objective. (3) Once inserted, an IUD can provide ongoing contraception for 5 years or more. A recent study suggested that women appear to have interest in “same-day” IUD insertion following unprotected intercourse, particularly better educated young women and those who had a prior unwanted pregnancy [15].

**Figure 1:**
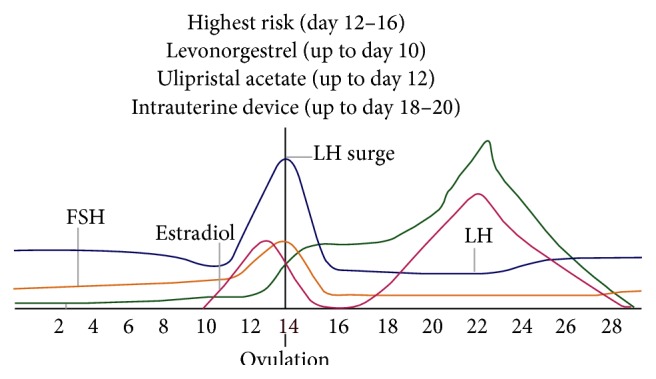
Fertility risk and window of action of different methods of emergency contraception.

Furthermore, IUDs are unaffected by BMI, timing of unprotected intercourse, or additional sex after IUD placement.  Figure 1 shows the window of action of different emergency contraceptive methods in relation to ovulation.

### 3.2. Insertion-Related Aspect

The IUD is an extremely safe method but requires placement by an appropriately trained health care provider. Placement may often be difficult and present a challenge especially in adolescent and nulliparous women or it is required by physicians with limited experience. In a study of women who were scheduled for IUD insertion for EC, 19% had a failed insertion, meaning that the provider was unable to insert the IUD and these women received oral EC instead [16]. There are however a number of practical points which could help in facilitating insertion. Information should be provided about the insertion procedure and measures taken to reduce discomfort associated with IUD insertion. Also information about the benefits and risks of IUD use should be given. Attention to comfort is very important as many women may refuse an IUD purely because of fear for pain. Insertion pain cannot be predicted. Ultrasonography does not give additional information to predict pain. According to Kaislasuo et al., dysmenorrhea is the only predictor of severe or intolerable insertion pain due to increased uterine/cervical contractility [17]. Utilizing means of sufficient analgesia, especially in nulliparous women, is important. Equally important is an appropriate patient-friendly setting accommodated with soft ambient light. Not to be forgotten is the use of a narrow and short speculum in young women to facilitate access to the cervix. A 1.0–1.5 cm wide speculum is sometimes necessary in young women. A too large speculum often causes more pain than the IUD insertion procedure. Listening to music during the procedure decreases procedural pain [18].

The injection of a small amount of lidocaine or mepivacaine in the anterior lip of the cervix before placement of (preferably) atraumatic forceps is a good habit, especially if the patient is anxious, or if slow closing of the Allis forceps causes pain, or prior to placement of a toothed forceps (e.g., Pozzi forceps). The use of a dental syringe with extrafine needle is highly practical and can also be used for local or locoregional anesthesia.

After disinfection and gentle straightening of the uterus, a “cotton swab test” (soaked in antiseptic solution) can be performed to test the tightness of the internal cervical os and to obtain information on pain sensation. If the test provokes severe pain, additional local anesthesia can be provided prior to sounding the uterus.

Many believe that the use of misoprostol greatly facilitates IUD insertion as it dilates the cervix [19]. We recommend the use of 200–400 *μ*g of misoprostol, orally or vaginally, 3 hours before IUD insertion. Others prefer to place the tablets vaginally in the posterior fornix the night before the procedure (9–12 hours before) but one hour sublingually before placement of the IUD may also be a good alternative (unpublished observations). Despite conflicting published data about the benefits of misoprostol, significant differences were found in nulliparous women between groups using misoprostol, 400 *μ*g vaginally, 4 hours before insertion, compared to placebo with less difficulty and less moderate-to-severe pain at IUD insertion [20]. It may be that the vaginal route could be preferable as plasma concentrations of misoprostol remain substantially higher than that when administered by the oral or sublingual routes. However, most women prefer the oral route to vaginal application [21]. It is recommended that a nonsteroidal analgesic should be added to reduce its prostaglandin-mediated side effects and uterine cramping. Some physicians claim that applying heat to the lower abdomen (using electric heating pad or a microwave-heated cherry seed pillow) may significantly reduce painful sensations [22]. If the patient is having severe discomfort with the insertion of the sound or requires cervical dilation, then the administration of a paracervical block or even conscious sedation (such as propofol or midazolam) can be used. However, this is rarely necessary and perhaps only in extremely anxious women.

### 3.3. Safety of Intrauterine Devices

The risk of pelvic infection, which may lead to infertility, ectopic pregnancy, and chronic pelvic pain, remains one of the major concerns of IUD providers as well as of women. There is good scientific evidence that the risk of pelvic inflammatory disease (PID) is not increased after the first month following insertion of the IUD. Investigations by WHO showed that the risk of PID is limited to the first 20 days after insertion [23]. Cervicitis at the time of insertion is not absolute contraindication and patients can be treated and the IUD is placed as the risk of developing pelvic inflammatory disease remains very low.

Many practitioners however remain concerned about PID, especially in higher risk populations. WHO suggests that the benefits of IUDs generally outweigh the risks in women of any age, whether parous or not, and that IUDs can be inserted in women younger than 20, provided that these women are at low risk of sexually transmitted infections. Rates of PID may vary between 0.6 and 1.6 per 1000 woman-years [23]. However, WHO also advises against the use of IUDs in women who have had PID in the previous 3 months [24]. ACOG recommends screening all adolescents at the time of or before IUD insertion. A practical solution is to test all high risk patients and to place the IUD on the same day. If the test results are positive, then treatment should be administered immediately (a single oral dose of 1 g of azithromycin or 2 × 100 mg of oral doxycycline for 7 days). Women who develop symptoms of PID after IUD insertion can be safely treated with antibiotics without removing the IUD.

### 3.4. Dimensional Aspects Related to IUD Use

Despite the fact that many women would select an IUD for EC if an IUD was proposed to them, the continuation rates in those who selected the IUD are rather poor. In a study conducted in the US using TCu380A (ParaGard), approximately 40% of IUDs were removed during the first year due to side effects indicating the need for newer IUD designs and better tolerated IUDs [25].

**Figure 2:**
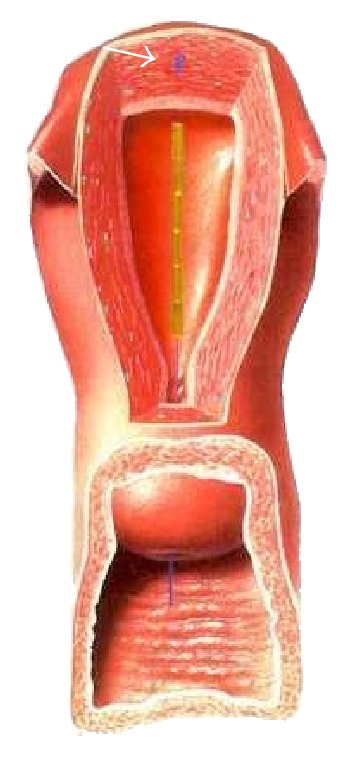
Illustration of the frameless GyneFix IUD anchored in the fundus of the uterus (see arrow). The anchoring knot is inserted in the fundus with a specially designed inserter.

**Figure 3:**
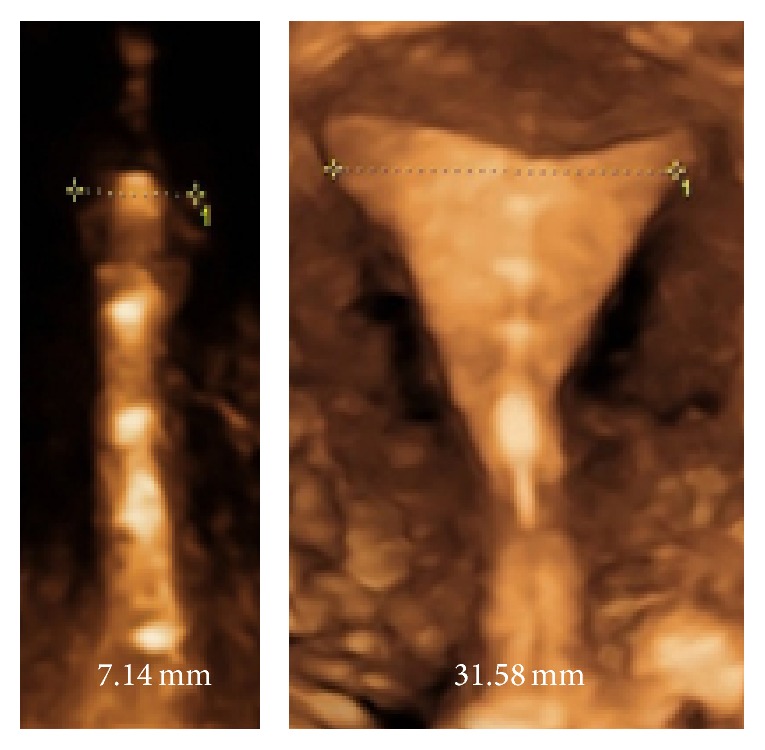
3D illuatration in two women fitted with a frameless IUD showing the disparity in width of the IUD which varies in these women between 7.14 and 31.58 mm.

Researchers have stressed the importance of an optimal interrelationship between the IUD and the uterine cavity for many decades in an attempt to have fewer side effects and greater acceptability [26]. They found that pain and abnormal bleeding during use of the IUD is related to the disparity between the size of the uterine cavity and that of the IUD. The width of the uterine cavity appeared to be most important in relation to IUD side effects. In a study in Finland conducted on 165 young nulliparous women, the uterine cavity width was measured with 3D ultrasound and found a* median* transverse fundal diameter of the uterine cavity of 24.4 mm. One hundred and one (62.7%) women had a transverse diameter at the fundus of less than 24.4 mm (Table 3). Thus, a very large segment of the female population have substantially smaller uterine widths. The smallest diameter observed in the study was 13.8 mm [27].  Figure 2 illustrates the frameless IUD inserted in the uterus and Figure 3 shows the disparity in cavity width in two nulliparous women.

The great disparity in size and shape of the uterine cavity in nulliparous women is shown in Table 1.

The mean transverse uterine cavity dimension in nulliparous women is far less than the length of the transverse arm of most conventional IUDs (e.g., ParaGard), which is 32 mm, resulting often in distortion, displacement, embedment, and expulsion of the IUD. In order to circumvent this spatial incompatibility, researchers adapted T-shaped IUDs. It was found that the fundal transverse dimension is of paramount importance with respect to IUD acceptance, as women tolerated the IUD much better [28]. An optimal IUD-cavity relationship also promotes IUD retention and stability while minimizing endometrial/myometrial trauma.

Frameless copper IUDs could be the optimal design from a dimensional point of view. The copper 330 frameless IUD has been used with good results in EC studies [6]. Due to its absence of a horizontal transverse arm and its flexibility, the device adapts to uterine cavities of every size and shape. These characteristics eliminate the ability of the uterus to exert expulsive forces on frameless IUDs, in contrast to that seen with the framed T-shape designed IUDs, and result in high efficacy, low expulsion rate, reduced bleeding, reduced or no pain complaints, long duration of action, and most importantly long-term comfort. The design characteristics of the frameless IUD would be attractive as a first choice method for many women and for young women requesting EC, especially for those with a small (e.g., nulliparous women) or distorted uterine cavity, and for women who have experienced problems with framed IUDs. The one-dimensional design of the frameless LNG-IUS explains its high acceptability and high continuation of use (over 90% at 5 years) [29, 30]. Figure 3 illustrates the dimensional compatibility even in women with very narrow uterine cavities.

The small size of the frameless IUD also results in a reduced effect on the amount of menstrual blood lost. It does not significantly increase menstrual blood loss, as may occur with framed IUDs due to its overall small size. This is important as heavy menstrual bleeding is the most common reason for IUD discontinuation.  Table 2 is a short list of questions which are relevant to patients requesting EC.

## 4. Conclusion

Adolescents and young nulliparous women are the groups that are most likely to be EC users. Yet, they are the least well-informed groups about EC in general and the use of an IUD for EC in particular. This paper offers practical information which may be helpful to choose the best option for women.  Figure 1 could be used as a poster for health care providers as well as women as it clearly visualizes the fertility risk and window of action of different methods of emergency contraception.

## Key Message Points

- In real life, IUDs are much more effective than the pill, contraceptive patch, and vaginal ring, especially in young women.
- The copper IUD is the most effective method for EC, significantly more effective than oral EC.
- A copper IUD can be inserted up to 5 days after unprotected intercourse or 5 days after the calculated ovulation day or anytime in the cycle if a high sensitivity pregnancy test is negative.
- Copper IUDs are effective regardless of overweight or obesity and frequency of intercourse in the cycle.
- IUDs provide long-term contraception but not all IUDs fit in young nulliparous and adolescent women.
- Women are interested in safe, effective, well-tolerated, and long-acting contraception; the frameless IUD would appeal to them as its acceptability is high.
- Clinicians who lack training for IUD insertion should refer women requesting an IUD in a timely manner.

## Figures and Tables

**Table 1:** Fundal transverse diameter (mm) in 165 Finnish nulliparous women [6].

	Range	50th percentile measure	No (%) under 50th percentile
Fundal width (mm)	13.8–35.0	24.4	101 (62.7)

**Table 2:** Short questionnaire to help select the EC method for the individual patient.

Question	Comment
(1) Which contraceptive method did you use up to now?	The pill, contraceptive patch, and the vaginal ring have a typical failure rate of 9% during the first year of use.
(2) When did your last menstrual period start?	Calculating the expected date of ovulation is important to select the EC method.
(3) When did you have unprotected sex?	All oral EC methods can be used up to day 10–12 of the menstrual cycle with preference for UPA close to ovulation. Oral ECs may not be safe 1 or 2 days before ovulation and are not effective after ovulation.
(4) Do you want to use a long-acting method of contraception?	An IUD should be the method of choice because of its high EC efficacy and ongoing protection.
(5) Do you have a stable relationship?	Women in a stable relationship have a low risk whilst women having sexual relations with different partners over the last month are at higher risk.
(6) Have you been treated for a sexually transmitted disease over the past 3 months?	IUD insertion may be performed immediately following screening tests and antibiotics should be prescribed if tests are positive.

**Table 3:** Uterine width measured by ultrasound in 165 nulliparous women. Note wide in uterine width as the high number of women with a uterine cavity less than 24 mm [28].

	Range	50th percentile measure	*N* (%) under 50th percentile
Fundal width (mm)	13.8–35.0 mm	24.4 mm	101 (62.7)
